# Community scientist program provides bi-directional communication and co-learning between researchers and community members

**DOI:** 10.1017/cts.2023.703

**Published:** 2023-12-21

**Authors:** Jessica Alvarado, Larkin L. Strong, Birnur Buzcu-Guven, Leonetta B. Thompson, Erica Cantu, Chelsea C. Carrier, Chiamaka D. Chukwu, Cassandra L. Harris, Luz K. Melendez, Crystal L. Roberson, Angela M. Ross, Sophia C. Russell, Pablo Sanchez, Amirali Tahanan, Blair C. Zdenek, Belinda M. Reininger, Lorna H. McNeill

**Affiliations:** 1 School of Health Professions, The University of Texas at Tyler, Tyler, TX, USA; 2 Department of Health Disparities Research, University of Texas MD Anderson Cancer Center, Houston, TX, USA; 3 UTHealth Houston School of Public Health Regional Campus at Brownsville, Brownsville, TX, USA; 4 UTHealth Houston McWilliams School of Biomedical Informatics, Houston, TX, USA; 5 UTHealth Houston, Houston, TX, USA

**Keywords:** Community engagement, community scientists, translational researchers, Texas, co-learning

## Abstract

Community involvement in research is key to translating science into practice, and new approaches to engaging community members in research design and implementation are needed. The Community Scientist Program, established at the MD Anderson Cancer Center in Houston in 2018 and expanded to two other Texas institutions in 2021, provides researchers with rapid feedback from community members on study feasibility and design, cultural appropriateness, participant recruitment, and research implementation. This paper aims to describe the Community Scientist Program and assess Community Scientists' and researchers' satisfaction with the program. We present the analysis of the data collected from 116 Community Scientists and 64 researchers who attended 100 feedback sessions, across three regions of Texas including Northeast Texas, Houston, and Rio Grande Valley between June 2018 and December 2022. Community Scientists stated that the feedback sessions increased their knowledge and changed their perception of research. All researchers (100%) were satisfied with the feedback and reported that it influenced their current and future research methods. Our evaluation demonstrates that the key features of the Community Scientist Program such as follow-up evaluations, effective bi-directional communication, and fair compensation transform how research is conducted and contribute to reducing health disparities.

## Introduction

Patient and community stakeholder engagement is increasingly recognized as critical for enhancing the relevance, quality, and benefits of research. The acknowledgment of the significance of community engagement in research hastens the translation of research findings and advances successful recruitment of clinical trial and community research participants [1,2]. Without a community-engaged research agenda that focuses on finding answers to both medical and public health questions, issues such as disproportionate access to health care, low-quality care, and elevated costs will persist [3]. With that in mind, grant programs such as the Patient-Centered Outcomes Research Institute require input from patients, caregivers, and stakeholders for their funded proposals, and many other granting agencies are following [4].

Previous community-based translational research efforts and community engagement studios have demonstrated the importance of community members and stakeholders in the formation and execution of research [1,2]. Modeled after the Community Engagement Studios of the Meharry Vanderbilt Community Engaged Research Core [5] and informed by existing successful programs [1,2], the Community Scientist Program (CSP) was established in 2018 at the University of Texas (UT) MD Anderson Cancer Center as part of the Center for Community-Engaged Translational Research and in collaboration with the UT Health Center for Clinical and Translational Sciences (CCTS). This program was designed to provide investigators with timely feedback from community and patient stakeholders on various aspects of research. Specifically, the stakeholders – Community Scientists – provide researchers with meaningful insights into proposed research projects, drawing from their lived experiences with cancer and/or a chronic illness.

The purpose of this paper is to describe the CSP and assess Community Scientists' and researchers' satisfaction with the program, while also evaluating the overall impact of the CSP on research feasibility, cultural appropriateness, research design, participant recruitment, and dissemination from the perspective of both Community Scientists and researchers. This work was determined to be non-human subjects research by the Institutional Review Boards at the University of Texas MD Anderson Cancer Center, The University of Texas at Tyler, and The UTHealth Brownsville Campus.

## Methods

### Description of the Community Scientist Program

The CSP was established to provide researchers with meaningful insights from local community members on their lived experiences with cancer and chronic illnesses, or cultural and social factors that impact health outcomes (e.g., African American cultural view). The CSP focuses on bi-directional communication and co-learning between researchers and community members and aims to improve public health and healthcare through community-engaged research. Through facilitated discussions called feedback sessions, designed to obtain community input about the research topic, community members, patients, and researchers are brought together to improve the relevance and quality of research and its overall outcome.

After three years of successful execution at MD Anderson, the program was expanded in 2021 to the University of Texas at Tyler Health Science Center in Northeast Texas and the UTHealth School of Public Health at Brownsville in the Rio Grande Valley with the support from a Clinical and Translational Science Award (CTSA) through the National Center for Advancing Translational Sciences [6]. This expansion resulted in researchers having access to urban, suburban, rural, and Spanish-speaking Community Scientists from various racial and ethnic backgrounds.

### Community Scientists

Community Scientists are patients, cancer survivors, people living with a chronic illness, caregivers, or members of racial and ethnic minority populations who provide feedback to researchers on how to improve their research to meet community needs. Community Scientists are recruited through various community outreach events and networks, tailored to each Texas region. Community members who are interested in being a Community Scientist complete an online application. This application form collects data on demographic characteristics, health status (including cancer survivors, and individuals with chronic illnesses), and caregiver background. Community members also indicate their availability for participating in orientation and training & feedback sessions and their health/research interests & experiences. Program staff review each application and schedule interviews with applicants to assess availability and fit for the program. Community members can be excluded from the program if they are directly involved in research, unavailable for training or feedback sessions, unresponsive to calls and emails, or if they do not have the characteristics and experiences that align with the researchers’ needs.

### Community Scientist Training Sessions

Individuals invited to become Community Scientists are required to participate in a virtual four-hour training session on topics surrounding the foundations of research (e.g., human subjects, research principles), the importance of community engagement in research, and how to engage with researchers. The training program includes a didactic component consisting of 30–45-minute lectures, as well as opportunities for interaction and hands-on experiences, such as discussions, role-playing, videos, and group exercises. Discussions and group exercises are initiated to discuss awareness of participants about their access to different resources. Videos are utilized to discuss the importance of community involvement in research. Role-playing and mock feedback sessions assist Community Scientists in understanding what to expect and how to provide feedback effectively. Upon completing the training, Community Scientists are then able to participate in feedback sessions.

#### Feedback sessions

Feedback sessions are one-hour facilitated sessions intended to gather community input on various aspects of research, such as feasibility, cultural appropriateness, study design, program implementation and/or dissemination, and participant recruitment. From 2018 to 2020, feedback sessions were conducted in person. However, in March 2020, the program transitioned to an entirely virtual delivery format in response to the coronavirus disease 2019 (COVID-19) pandemic and has since continued with this mode of delivery. Currently, feedback sessions are conducted via Zoom or Webex and consist of a 15–20-minute period during which researchers present their study and ask Community Scientists questions. To promote active listening and engagement for researchers, the program staff facilitate the discussion and take notes. This allows researchers to fully participate in the conversation without the need to take notes. At the end of the session, the program staff provide researchers with a comprehensive report of the discussion.

Feedback sessions can be arranged to accommodate researchers’ requirements to include Community Scientists from a single region or all three program regions. These sessions are scheduled to take place 1–2 times a month across three program regions. Community Scientists are compensated with a $25 electronic gift card for their participation in feedback sessions.

#### Researchers

Researchers can request a feedback session by completing an online form that asks for information about themselves, research topic and feedback they are interested in, the funding agency, Community Scientist characteristics required for their research (e.g., cancer survivors, persons with chronic illnesses, etc.), and region(s) of interest. There is no cost to researchers to participate in a feedback session.

The CSP staff host a preparation meeting with researchers to provide an overview of the CSP, the format of the feedback sessions, a feedback presentation template that encourages the use of plain language and engagement format (e.g., overview, study design, problem, questions), and an opportunity to share the required Community Scientist characteristics with the program staff. The program staff identifies Community Scientists whose characteristics and experiences align with the researchers’ needs and invites them to participate in the feedback session.

#### Evaluations

After each feedback session, the program staff sends brief evaluations to Community Scientists to assess their level of satisfaction with the sessions, the quality of the researchers’ presentation, and the perceived value of the Community Scientists’ feedback to the researchers and the study.

At the end of each calendar year, Community Scientists are requested to complete another survey to measure their satisfaction with the overall program and individual sessions, as well as to evaluate compensation, communication from staff, and the program’s impact on their understanding of research in general.

Researchers are requested to complete electronic evaluations immediately after the feedback session, as well as at 6- and 12-month marks. These evaluations aim to measure their overall satisfaction with the session, scheduling and communication, the impact of Community Scientists’ feedback on research methods, contributions of Community Scientists to the research project, and planned project changes based on the feedback.

#### Data quality control

The data for this work were obtained through web-based surveys, which were completed by Community Scientists and researchers who participated in feedback sessions. All data were collected and managed using REDCap (Research Electronic Data Capture) tools hosted at MD Anderson [7,8]. Data quality control involves a series of procedures that guarantee the reliability of the collected data. To ensure the highest level of data quality, we corrected and completed all discrepant data, missing values, and incorrect or implausible responses, which were logically inconsistent with other data present in the database, by contacting Community Scientists.

### Data Analysis

We conducted descriptive analysis of the quantitative data in the database and generated summary statistics such as frequencies and percentages. All descriptive statistics were obtained using SAS 9.4 [9].

## Results

A total of 116 people across the three regions completed the training since the program’s inception in 2018. Most Community Scientists were women (78.1%), had completed some college (86.8%), and were employed (77%) (Table 1). Over half (56.9%) were based in Houston, followed by Rio Grande Valley (27.6%), and Northeast Texas (15.5%). Approximately half (46.4%) were Hispanic/Latino, 37.5% were non-Hispanic Black, and 14.3% were non-Hispanic White; however, race/ethnicity varied considerably by region, reflecting the different demographics of these areas. Approximately 25% of the Community Scientists were cancer survivors, and 43.2% had served as caregivers for cancer patients and/or individuals with a chronic disease.

**Table 1.** Characteristics of community scientists – overall and by region

	Total (*N* = 116) *N* (%)	Houston (*N* = 66) *N* (%)	Northeast Texas (*N* = 18) *N* (%)	Rio Grande Valley (*N* = 32) *N* (%)
Women^a^	89 (78.1)	49 (75.4)	16 (94.1)	24 (75.0)
Age >= 40 years^b^	59 (68.6)	24 (52.2)	5 (38.5)	22 (68.8)
Race/ethnicity^c^				
Non-Hispanic Black	42 (37.5)	34 (53.1)	8 (50.0)	0 (0)
Hispanic/Latino	52 (46.4)	19 (29.7)	2 (12.5)	31 (96.9)
Non-Hispanic White	16 (14.3)	9 (14.1)	6 (37.5)	1 (3.1)
Asian/American Indian/ Native Hawaiian, Alaskan/Other Pacific Islander	2 (1.8)	2 (3.1)	0 (0)	0 (0)
Education^a^				
≤High School grad/GED	15 (13.2)	4 (6.2)	1 (5.9)	10 (31.3)
Some college/Associate’s	28 (24.6)	13 (20.0)	5 (29.4)	10 (31.3)
Bachelor’s degree	27 (23.7)	18 (27.7)	3 (17.7)	6 (18.8)
>Bachelor’s degree	44 (38.6)	30 (46.2)	8 (47.1)	6 (18.8)
Cancer Survivor^d^	28 (24.8)	23 (35.9)	1 (5.9)	4 (12.5)
Caregiver^e^	41 (43.2)	24 (52.2)	7 (41.2)	10 (31.3)
Employed^d^	87 (77.0)	43 (66.2)	14 (82.4)	30 (96.8)

a 2 respondents did not answer.

b 30 respondents did not answer; question changed in 2022.

c 4 respondents did not answer.

d 3 respondents did not answer.

e 21 respondents did not answer; question added in 2020.

### Program Evaluation by Community Scientists

Between June 2018 and December 2022, 100 feedback sessions were conducted with an average of 8.4 Community Scientists per session (range 3–18). Ten sessions were conducted in Spanish, all in Rio Grande Valley. The number of feedback sessions doubled in 2021 with the program’s expansion to Northeast Texas and Rio Grande Valley (12 vs 24). A total of 753 feedback session evaluations were completed by 97 out of 116 Community Scientists who completed training. In general, evaluations were overwhelmingly positive (Table 2). Nearly all Community Scientists expressed satisfaction with the implementation of feedback sessions (e.g., time management, scheduling, communication), stated that they would participate in a feedback session again, and felt that their feedback was valued and useful to the study. Community Scientists’ perceptions of their contributions to research varied (Table 2). The most frequently cited contributions included enhancing researchers’ understanding of the community (52.2%), increasing their sensitivity to the community (40.4%), and offering feedback on the feasibility of the study (39.8%).

**Table 2.** Evaluations of feedback sessions by community scientists and their perceived contributions to research

Feedback session evaluations (*N* = 753)	Strongly agree/agree *N* (%)
The scheduling/communications for the feedback session was handled in a timely and efficient manner	736 (98.4)
The allotted time for the feedback session was sufficient	668 (88.8)
I felt my feedback was valued*	435 (98.9)
The feedback session facilitator managed the allotted time in order to address my questions and comments	741 (98.7)
I was satisfied with the feedback session	741 (98.5)
The feedback session process was worth my time	738 (98.8)
The feedback I provided will improve the research project	719 (98.1)
The researcher’s presentation gave me enough information to provide appropriate feedback	730 (97.3)
	Yes N(%)
Would you participate in a feedback session again?	738 (99.9)

Community scientists’ perceived contributions to the research project (*N* = 753)	Strongly agree/agree *N* (%)
Increased researcher understanding of the community	393 (52.2)
Increased researcher sensitivity to the community	304 (40.4)
Provided feedback on the feasibility of the project	300 (39.8)
Provided feedback on the appropriateness of the project	243 (32.3)
Ideas on recruiting research participants	271 (36)
Ideas on how to inform the community about the project	288 (38.3)
Ideas on how to use results of project to benefit the community	165 (21.9)
Other	62 (8.2)

* 318 were missing because question was added in 2021.

The end-of-year evaluations were completed in 2019, 2020, 2021, and 2022 by 100%, 76%, 33%, and 56% of the Community Scientists who were asked to complete the evaluation, respectively. The decrease in the response rate can be attributed to the increase in the number of Community Scientists and changes in the delivery method over the years. Moreover, the end-of-year evaluation was only administered to those who attended the New Year’s celebration meetings, which have ranged from in-person (2019 and 2020) to virtual (2021) formats. The end-of-year evaluations showed that similar to feedback session evaluations, Community Scientists had a very positive assessment of the program overall (Table 3). Nearly all Community Scientists who responded expressed satisfaction with how the program was conducted, including the communication from staff, the application process, and the training session. Although a smaller percentage, the vast majority of Community Scientists felt that there was enough time for discussions (83.1%) and that their input was valuable to the research (80.9%). In addition, most Community Scientists (95.5%) stated that the program had increased their knowledge of research, and over two-thirds expressed that their perception of research had changed after participating in the program.

**Table 3.** End-of-year evaluations by community scientists (2019–2023) (*N* = 89)

	Very Satisfactory/ Satisfactory *N* (%)
Overall, how would you rate the Community Scientist Program?	88 (98.9)
Overall, how would you rate the Community Scientist Program Feedback Sessions?	87 (98.8)
Overall, how would you rate the communication you received from the Community Scientist Program staff?	88 (98.9)
Overall, how would you rate the training you received for the Community Scientist Program?^a^	41 (97.6)
How would you rate the registration process for the feedback sessions?	85 (95.5)
How would you rate the application process for the Community Scientist Program?	86 (96.6)

	All the Time/Most of the Time *N* (%)
How often do you feel there is enough time for discussion during the feedback sessions?	74 (83.1)
How often do you feel like your input at the feedback sessions was beneficial to the researchers who presented?	72 (80.9)
	Yes N (%)
Did the program increase your knowledge of research enough that you felt comfortable sharing/teaching other people what you learned?	85 (95.5)
Have you shared what you learned from the Community Scientist Program with friends and/or family?	66 (74.2)
Did your perception of research change after participating in the Community Scientist program?	59 (69.3)

a Question was no longer asked in 2022.

Open-ended responses to follow-up questions regarding how Community Scientists’ perceptions had changed after feedback sessions were classified into three primary categories:

1) Increased understanding that research is conducted to benefit patients/communities.

“*I thought research was impersonal and only data-driven in order to meet funding deadlines or requirements. What I have found is that those who propose research do it painstakingly in order to answer important questions that plague the community or the patients we serve*.” (Community Scientist, female, African American)

2) A recognition and sense of empowerment regarding the importance of community/lay input into the research process.

“*It is great to know that individuals that are part of the community are able to impact the direction of a research based on the professional perception of what will create a successful research activity.*” (Community Scientist, female, White)

3) Greater insight into what research encompasses including the breadth and diversity of research studies.

“*Participation in the Community Scientist Program caused me to view research as more human and not just lab coats and test tubes.*” (Community Scientist, female, African American)

### Program Evaluation by Researchers

Researchers are requested to complete evaluation forms immediately following the feedback sessions and again 6 and 12 months later. Between March 2018 and December 2022, a total of 89 feedback session evaluations (after the session) were completed with 64 unique responses. All (100%) researchers expressed satisfaction with the feedback sessions and felt that the sessions were worth their time (Table 4). When asked about their thoughts before and after the session, nearly all researchers reported feeling more prepared to engage (98.3%) and collaborate (100%) with community members and recognizing the value of community collaboration and input in research (95%) after the feedback session. The items that the researchers plan to change as a result of their feedback session varied, with the majority stating that they would revise their recruitment/retention strategies (44.9%), use less technical/medical jargon (29.2%), increase the level of community/patient engagement in research activities (27.0%), and modify research design (22.5%).

**Table 4.** Feedback session evaluations by researchers

Variable	Strongly agree/Agree *N* (%)
I was satisfied with the Community Scientist Feedback Session	89 (100)
Presenting my study at the Community Scientist Feedback Session was worth my time^a^	88 (100)

	Somewhat/Very *N* (%)
How prepared did you feel to engage with community members, patients and/or stakeholders BEFORE collaborating with the Community Scientist Program?^b^	54 (90)
How prepared do you feel to engage with community members, patients and/or stakeholders NOW?^b^	59 (98.3)
How valuable did you see community collaboration and/or input in research BEFORE collaborating with the Community Scientist Program?^b^	57 (95)
How valuable do you see community collaboration and/or input in research NOW?^b^	60 (100)
	Yes *N* (%)
Has your perception about the role of patient, survivor or community groups in research changed as a result of the Community Scientist Feedback Session?^c^	11 (12.6)

What, if anything, do you plan to change as a result of the feedback you received from the Community Scientist Feedback Session? Check all that apply	*N* (%)
Research question	9 (10.1)
Research design	20 (22.5)
Level of community/patient engagement in research activities	24 (27.0)
Recruitment/retention strategies	40 (44.9)
Consent process	5 (5.6)
Data collection	13 (14.6)
Data interpretation	5 (5.6)
Dissemination	18 (20.2)
Change in number of questions (i.e., survey items)	13 (14.6)
More patient-centered questions	12 (13.5)
Less technical/medical jargon	26 (29.2)
More culturally relevant questions	16 (18.0)
Other	27 (30.3)
I do not intend to change anything	1 (1.1)

In what ways has your experience with the Community Scientist Program influenced your current or future research methods? (*n* = 89)	*N* (%)
Made connections with patients, community members and/or stakeholders	26 (29.2)
Learned new methods for recruiting participants	23 (25.8)
Learned community-engaged approaches to conducting research	19 (21.4)
Learned ways to adapt research study materials to be culturally and/or linguistically appropriate for the target audience	44 (49.4)
Got ideas for additional study aims	15 (16.9)
Received feedback on the appeal of study incentives	21 (23.6)
Increased understanding of the target audience	35 (39.3)
Made major changes to the study design (i.e. inclusion/exclusion criteria, data collection methods, study timeline, etc.)	7 (7.9)
Other	9 (10.1)

What do you feel were the Community Scientists' contributions to your research project? (*n* = 89)	*N* (%)
Increased my understanding of the community	53 (59.6)
Increased my sensitivity to the community	45 (50.6)
Provided feedback on the feasibility of the study/project	60 (67.4)
Provided feedback on the appropriateness of the study/project	64 (60.7)
Ideas on recruiting research participants	40 (44.9)
Ideas on how to inform the community about the study/project	50 (56.2)
Ideas on how to use results of study/project to benefit the community	23 (25.8)
Other	15 (16.9)

a 1 participant did not answer.

b 29 participants did not answer.

c 2 participants did not answer.

The CSP had a significant impact on the researchers’ current and future research methods in three major ways: they learned how to adapt study materials to be culturally and/or linguistically appropriate for the target audience (49.4%), gained a deeper understanding of the target audience (39.3%), and learned how to establish connections with patients, community members, and other stakeholders (29.2%) (Table 4).

The researchers felt that Community Scientists helped increase their understanding of the community (59.6%), sensitivity to the community’s characteristics (50.6%), as well as offered feedback on the feasibility (67.4%) and appropriateness (60.7%) of the study (Table 4).

## Discussion

There is a vast opportunity to advance scientific research by learning from the experiences of community stakeholders. Our evaluation of the CSP indicates that both Community Scientists and researchers found the feedback sessions to be satisfactory, highlighting the effectiveness of bi-directional communication and co-learning among session participants. Researchers were able to share relevant information about their research during feedback sessions, enabling Community Scientists to provide valuable feedback informed by their own lived experiences.

The hands-on virtual training for newly recruited Community Scientists is vital to the success of feedback sessions and the program. It builds the capacity and skills of community members to actively participate in the research process. It can empower Community Scientists to critically appraise research findings, interpret study results, and effectively communicate these research findings back to the community, which can contribute to community health literacy and promote evidence-based decision-making. This ensures that the research process is community-driven, relevant, and responsive to the community through quality feedback.

An important strength of the CSP is the inclusion of a follow-up survey conducted with both community members and researchers who participated in the feedback sessions. Previous community engagement studio models have also yielded positive outcomes when researchers actively sought feedback from stakeholders. Joosten *et al*. [1] reported that 100% of researchers expressed a high likelihood of requesting another community engagement studio in the future and that participating in the community engagement studio increased researchers’ appreciation for patient input in research and their understanding of how to overcome barriers to participation. Another article [10] described how community stakeholders believed that their feedback would improve research, making it a worthwhile investment of their time. The CSP, modeled after the community engagement studios, also demonstrates that community engagement in research benefits researchers and local communities involved.

The CSP represents one form of community engagement, which spans a continuum of community involvement ranging from none to community-driven or community-led [11]. By engaging with stakeholders to facilitate community input into the research process, the CSP can be at the level of “community consultation.” The program is not intended to replace other forms of community engagement; rather, it can be viewed as complementary. Importantly, as this work demonstrates, the program represents a valuable approach for sharing the value and benefits of community engagement with researchers in an expedient manner.

Themes developed from our findings – co-learning, bi-directional communication, and building capacity– continue to support the community engagement model. Co-learning and bi-directional communication facilitate reciprocal knowledge transfer between community members and researchers. For example, Community Scientists provide feedback and access to a wide social network, while researchers share opportunities to engage in conversations that prioritize health disparities in the best interest of the community. Another theme revealed from the data is the realization that conducting research alone is insufficient to make a change; both Community Scientists and researchers should commit, through joint ownership, to disseminate the findings and adopt the best practices resulting from the research. The feedback process for both Community Scientists and researchers involves capacity building, which is facilitated by providing culturally and linguistically appropriate interventions. This emerges as a key theme in the Community Scientists’ perspectives regarding their contributions to research studies.

The shift to an all-virtual delivery format for Community Scientist training and feedback sessions in response to the COVID-19 pandemic has yielded positive results, as we have consistently maintained an average of 8.4 participants in our monthly feedback sessions. The virtual format enables both community members and researchers to participate from any location if they have Internet access and a camera available. Since some Community Scientists are caregivers or patients, we have discovered that the virtual format significantly simplifies their involvement in the program. In addition, the virtual format enables us to successfully expand the program to other regions, including the Rio Grande Valley and Northeast Texas. The expansion into these regions offers opportunities to connect with a broader social network, encompassing Spanish-speaking communities.

### Limitations

Currently, Community Scientists are not receiving any data or information from researchers regarding how their feedback has impacted the project. However, it is crucial to recognize that sharing research findings with the community increases trust, bi-directional communication, and power dynamics within the research process [3]. To overcome this limitation, we plan to implement research update sessions, where we will invite previous researchers to share their findings with Community Scientists and provide updates on their research.

Although we recruit Community Scientists who are diverse and representative of their communities, it is important to acknowledge that self-selecting volunteers may not always fully reflect the perspectives and experiences of their respective communities. Instead, they may only share their own opinions. To address this limitation, we will continue to enhance our recruitment strategies in the CSP by connecting with public health agencies within our regions and attending community events to encourage participation from diverse community members.

Another limitation identified in the data is the presence of gender bias in participation. We observed that women are more likely to get involved in community engagement opportunities than men. This disparity could be attributed to a greater representation of women serving as liaisons for their respective community organizations (churches, advocacy groups, etc.) or their increased availability to participate in trainings and feedback sessions, which were scheduled during typical working hours. Regarding relevance of the research, the observed gender bias in participation could be perceived as a barrier. When women are overrepresented in community engagement opportunities, there is a risk that research findings might be less applicable to the broader community which could potentially affect the uptake and utilization of research findings in the community. It is important to address this limitation by developing strategies to increase diversity and representation among community members. These strategies may include targeted recruitment, flexible schedules for training and feedback sessions, and inclusive communication of research topics and findings to all members of the community, regardless of gender or other demographic factors. Additionally, addressing any cultural or societal norms that may contribute to gender bias in participation should be considered to promote inclusivity and diversity in community-based research efforts.

## Conclusions

This paper describes an approach intended to help create a deeper connection between researchers and communities and enhance translational research. We found that follow-up evaluations, effective bi-directional communication, and fair compensation can transform how research is conducted and translated to improve health disparities. The program is continuously evolving based on input from Community Scientists and researchers to maximize its impact. We are collecting data to highlight research advances resulting from community input and to assess how this program has strengthened community relationships and reduced social determinants of health, ultimately fostering practice-changing achievements and community development. Owing to the CSP, researchers submitting protocols are now directed to Community Scientist panels; the e-Protocol system includes a prompt about the program for human subjects research; pilot grants and training for new researchers involve learning about the CSP.
